# The effect of pandemic prevalence on the reported efficacy of SARS-CoV-2 vaccines

**DOI:** 10.1371/journal.pone.0266271

**Published:** 2022-04-05

**Authors:** Rajeev Sharma, Abhijith Anand

**Affiliations:** 1 Waikato Management School, University of Waikato, Hamilton, Waikato, New Zealand; 2 Department of Information Systems, Walton College of Business, University of Arkansas, Fayetteville, Arkansas, United States of America; University of Hail, SAUDI ARABIA

## Abstract

The efficacy of SARS-CoV-2 vaccines reported in Phase 3 trials varies from ~45% to ~95%. This study tests the hypothesis that the observed variation in efficacy of SARS-CoV-2 vaccine candidates can be explained by the prevalence of the COVID-19 pandemic at trial sites. To test the proposed hypothesis, we conducted a systematic search following PRISMA guidelines. Our search resulted in 8 vaccine candidates that had reported efficacy data from a total of 20 Phase 3 trials, representing a total of 221,968 subjects, 453 infections across the vaccinated groups and 1,554 infections across the placebo groups. We use meta-regression models to analyse the potential associations between prevalence of COVID-19 pandemic at trial sites and efficacy of the reported SARS-CoV2 vaccines. The overall estimate of the risk-ratio is 0.24 (95% CI, 0.17–0.34, p ≤ 0.01), with a high degree of heterogeneity (τ^2^ = 0.50, I^2^ = 88.73%). Our meta-regression analysis with pandemic prevalence as the predictor explains almost half the variance in risk ratios across trials (R^2^ = 49.06%, p ≤ 0.01). This study finds that efficacy of SARS-CoV-2 vaccines reported in Phase 3 trial declines as pandemic prevalence at trial sites increases. Trials conducted in locations with low pandemic prevalence reported higher efficacies as compared to trials conducted in high pandemic prevalence locations.

## Introduction

The SARS-CoV-2 pandemic has generated an unprecedented effort towards developing vaccines to halt the pandemic. A number of vaccines are under development, many have reported results of Phase 3 trials, and many have received emergency approval too. Vaccine efficacies reported across Phase 3 trials range from 45% to 96% [1–5], see Table 1. Efficacy results have influenced decisions in critical areas, including those related to patient care, public policy, and individual decisions leading to vaccine hesitancy [6–9]. At this stage of the vaccine development and emergency use authorization processes, it is important to analyse the available data and account for the effect of potential sources of heterogeneity on vaccine efficacy.

**Table 1:** Characteristics of phase 3 trials of SARS-CoV-2 vaccine candidates reporting efficacy.

Vaccine candidate	Number of subjects in vaccine group	Number of infections in vaccine group	Number of subjects in placebo group	Number of infections in placebo group	Reported efficacy (%)	Source
AstraZeneca AZD1222, Brazil (SD/SD)	2063	12	2025	33	64.2	Voysey et al (2021) [10]
AstraZeneca^a^ AZD1222- US, Chile and Peru	21633	62	10816	128	76	AstraZeneca (March 25, 2021) [11]
AstraZeneca AZD1222, UK (LD/SD)	1367	3	1374	30	90	Voysey et al (2021) [10]
AstraZeneca AZD1222, UK (SD/SD)	2377	15	2430	38	60.3	Voysey et al (2021) [10]
Bharat Biotech, COVAXIN, India	12900	7	12900	36	80.6	Bharat Biotech (March 3, 2021) [12]
Gamaleya rAd26/rAd5, Russia	14094	13	4601	47	91.1	Logunov et al (2021) [13]
Janssen JNJ-78436735, Argentina	1399	8	1409	30	73.3	Janssen Biotech Inc. (Feb 26, 2021) [14]
Janssen JNJ-78436735, Brazil	3370	39	3355	114	66.2	Janssen Biotech Inc. (Feb 26, 2021) [14]
Janssen JNJ-78436735, Chile	531	2	540	4	49.6	Janssen Biotech Inc. (Feb 26, 2021) [14]
Janssen JNJ-78436735, Columbia	1845	22	1858	62	64.7	Janssen Biotech Inc. (Feb 26, 2021) [14]
Janssen JNJ-78436735, Mexico	206	1	220	0	-	Janssen Biotech Inc. (Feb 26, 2021) [14]
Janssen JNJ-78436735, Peru	571	7	580	13	45.3	Janssen Biotech Inc. (Feb 26, 2021) [14]
Janssen JNJ-78436735, South Africa	2473	43	2496	90	52	Janssen Biotech Inc. (Feb 26, 2021) [14]
Janssen JNJ-78436735, United States	9119	51	9086	196	74.4	Janssen Biotech Inc. (Feb 26, 2021) [14]
Moderna mRNA-1273, US	14134	11	14073	185	94.1	Baden et al. (2021) [15]
Novavax NVX-CoV2373, South Africa	2206	51	2200	96	48.6	Novavax (March 11, 2021) [16], Novavax (January 28, 2021) [17], Gregory (February 2, 2021) [18]
Novavax NVX-CoV2373, UK	7016	10	7033	96	96.4	Novavax (March 11, 2021) [16], Novavax (January 28, 2021) [17], Gregory (February 2, 2021) [18]
Pfizer/BioNTech BNT162b2, US	18198	8	18325	162	95	Polack et al. (2020) [19]
Sinovac CoronaVac, Brazil	4953	85	4870	168	50.65	Sinovac [20, 21]
Sinovac CoronaVac, Turkey	752	3	570	26	91.25	Sinovac [20, 21]

Note: All reported efficacies are taken from the respective source documents (see column ‘Source’). Efficacy for Janssen’s Mexico trial cannot be computed as there are zero infections in the placebo group. Endpoints for efficacy calculations vary across trials but are comparable, viz. 7 days after the second dose for Novavax and Pfizer/BioNTech, 14 days after the second dose for AstraZeneca, Bharat Biotech, Moderna, and Sinovac, 21 days after the first dose of the two-dose Gamaleya vaccine, and 21 days after the first dose of the one-dose Janssen vaccine (S3 Table in S1 File).

^a^ For its US trial, AstraZeneca reports efficacy, total number of participants, split ratio between vaccinated and placebo groups, and the total number of infections. The infected numbers in each group were not reported, however, the data provided by AstraZeneca was sufficient for those numbers to be computed.

Differences in vaccine efficacies reported across trials could be due to various sources of heterogeneity between trials [18, 22, 23]. An important source of between-trial heterogeneity that could explain the differences in the efficacies observed across trials is the prevalence of the pandemic in the populations where the trials were conducted [14, 18, 22, 23]. Prevalence (or, test positivity ratio) refers to the proportion of the population that had tested positive to the SARS-CoV-2 virus at a trial location during the course of the trial [24]. The prevalence of the SARS-CoV-2 pandemic has varied across locations and over time. As an example, the prevalence reported in national testing programs has varied from near zero in New Zealand to over 40% in countries such as Mexico and Argentina [25]. Prevalence within countries has also varied over time. For instance, prevalence in the US has varied from a low of 4.0%-5.5% over June 2020 to a high of 10.6%-13.1% over December 2020 [25].

Variations in vaccine efficacy under different prevalence conditions have been reported previously in Phase 3 trials of other vaccines too [23]. For instance, in a Phase 3 trial of the malaria vaccine, RTS,S/AS01_E_, Kaslow [23] reports that “across 11 clinical research sites in seven African countries”, vaccine efficacies were reported to vary between 83.0% and 44%. Importantly, vaccine efficacy was found to decrease with the prevalence of the malaria virus across the trial sites [23]. Similarly, the European Medicines Agency, in its regulatory evaluation of the RTS,S/AS01_E_ malaria vaccine, observed that vaccine efficacy “tends to be lower in high transmission areas” [26, 27]. Kaslow [23] further reports that the efficacies of two rotavirus vaccines, RV1 and RV5, have been observed to fall with prevalence of the rotavirus infection. A similar pattern has been reported for the SARS-CoV-2 vaccine candidates also [28].

Variations in vaccine efficacy have been attributed to the presence of mutant strains of a virus, among other factors [29, 30]. Genetic mutations and antigenic drifts in a focal virus can lead to an antigenic mismatch between the vaccine and the target antigen, resulting in a failure of a body’s vaccine-induced anamnestic response and leading to a greater number of breakthrough infections in the vaccine arm of a Phase 3 trial [26, 29–33]. Such breakthrough infections have been reported for individuals vaccinated against the SARS-CoV-2 virus as well [28, 30, 34]. Vaccine efficacy, by definition, falls, *ceteris paribus*, when there are more breakthrough infections, i.e. a greater number of subjects in the vaccinated group of a trial getting infected. Consequently, as mutant strains generate a greater number of breakthrough infections, vaccine efficacy is likely to decrease in the presence of mutant strains. Genetic mutations and antigenic drift in a focal virus can also lead to higher prevalence. Mutant strains often possess additional paths, as well as a stronger ability, to infect human cells [29, 30]. Those abilities render the mutant strains more transmissible, leading to a higher prevalence [29, 30]. The Centre for Disease Control [28] reports that in the case of COVID-19 vaccine breakthrough infections, “The proportion of reported vaccine breakthrough infections attributed to variants of concern has also been similar to the proportion of these variants circulating throughout the United States.”

As mutations and antigenic drifts are responsible for both higher prevalence as well as lower efficacy, we hypothesize that *Phase 3 trials of SARS-CoV-2 vaccines carried out in high prevalence areas are likely to report lower efficacies*.

## Methods

### Search strategy and selection criteria

This study is a systematic review and meta-analysis of the efficacy data reported in Phase 3 trials of SARS-CoV-2 vaccine candidates. The meta-analysis reports an overall summary estimate of the risk-ratio and conducts a meta-regression to test the effect of between-trial differences in prevalence of the SARS-CoV-2 pandemic on efficacies reported across trial sites. The study was registered at PROSPERO (Registration Number CRD42021243121).

Vaccine candidates were included in this study if they reported efficacy results from Phase 3 randomised controlled trials. Results of Phase 3 trials reported till March 31, 2021 were included in the analysis. Phase 3 clinical trials of vaccine candidates are being conducted by the vaccine manufacturers only. Accordingly, we started our search by identifying vaccine candidates that had registered Phase 3 trials, following PRISMA guidelines. Given the high level of interest in the development of SARS-CoV-2 vaccines, such information is widely shared by the manufacturers and tracked by multiple reliable sources [1–3]. In particular, the WHO maintains a tracker database that compiles detailed information on the SARS-CoV-2 vaccine candidate landscape, tracks vaccine candidates in development, and regularly updates progress on registered trials that are underway: “*To ensure the latest information is available*, *the landscape will be updated twice a week (Tuesday and Friday*, *17*:*00 CET) by searching*, *gathering and cross-checking data from multiple sources such as the Cochrane vaccine mapping tool*, *PubMed*, *ClinicalTrials*.*gov*, *WHO ICTRP and from a network of researchers and industry for new candidate vaccines by screening registered trials for clinical information*. *Where data is missing*, *… we supplement information gathered from press or public releases*” [1]. Similar information is compiled independently by McGill University’s [2] and the LSHTM’s Vaccine Centre’s [3] COVID19 Vaccine Tracker websites. Given the rigorous process followed by those websites, which includes searching of key databases such as *PubMed* and *ClinicalTrials*.*gov*, we relied on those sources to identify vaccine candidates that had progressed to Phase 3 trials.

The data on vaccine candidates from the three websites was aggregated by RS to compile a master list of vaccine candidates (S1 Table in S1 File). A total of 26 unique vaccine candidates that had progressed to Phase 3 trials were identified. There was 100% concordance between the three tracker websites regarding vaccine candidates currently in Phase 3 trials (S1 Table in S1 File). RS then searched the information available on the three tracker websites, and *ClinicalTrials*.*gov* to identify the start dates of the Phase 3 trials for each vaccine candidate. Given the time lag between the start of Phase 3 trials and the reporting of efficacy results, trials that had not started by November 1, 2020 were excluded from a further search of results of Phase 3 trials. That left a total of 11 vaccine candidates to be searched for the results of efficacy data from Phase 3 trials. AA independently validated every step of the search and the master list of vaccine candidates through reference to additional sources (S1 Table in S1 File). No discrepancies were located, and no additional vaccine candidates were located.

**Fig 1:**
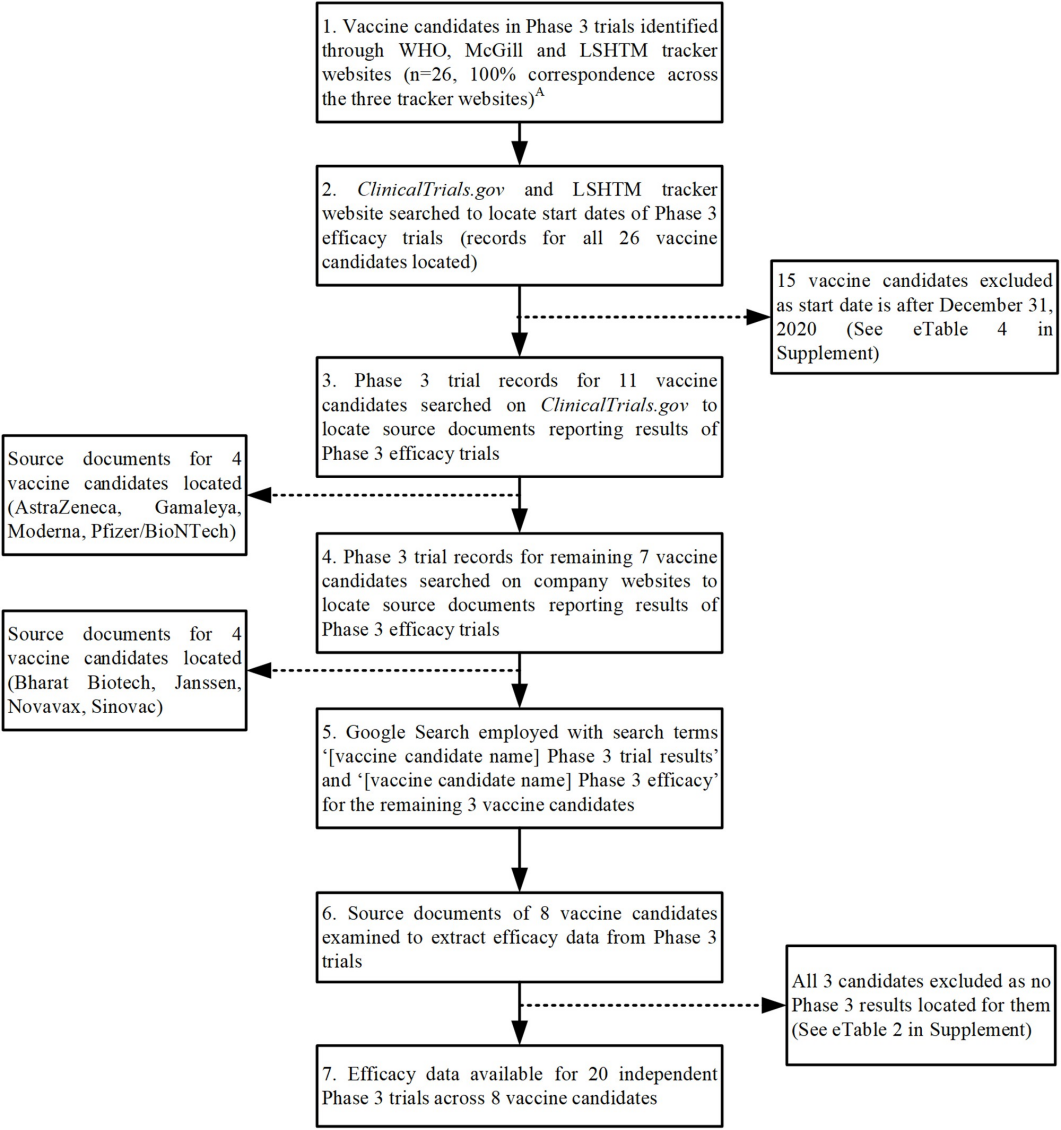
Study selection. This shows the step-wise process of study selection and pre-specified inclusion and exclusion criteria.

RS then searched for original sources where efficacy results of the Phase 3 trials were reported. The progress of vaccine candidates through various trial stages is also of intense public interest and receives unprecedented public scrutiny [1–3]. RS then located links to Phase 3 registered trials from all three tracker websites. Source publications reporting efficacy data for four vaccine candidates were identified on *ClinicalTrials*.*gov* (AstraZeneca, Gamaleya, Moderna, and Pfizer, see Fig 1). RS then visited the websites of the remaining vaccine manufacturers, which yielded source documents for another four vaccine candidates (Bharat Biotech, Janssen, Novavax, and Sinovac). No efficacy data could be located for the other 3 vaccine candidates, which are from Sinopharm and CanSino, who have chosen not to publicly report the data prior to the cut-off date for inclusion in this study [35] (S2 Table in S1 File).

The search identified 8 vaccine candidates that had reported efficacy data. Table 1 lists the efficacy-related data from 20 Phase 3 trials reported by those 8 vaccine candidates, viz. AstraZeneca AZD1222 (4 trials), Bharat Biotech Covaxin, Gamaleya rAd26/rAd5, Janssen JNJ-78436735 (8 trials), Moderna mRNA-1273, Novavax NVX-CoV2373 (2 trials), Pfizer/BioNTech BNT162b2, and Sinovac CoronaVac (2 trials). Additional details regarding the trials are reported in S3 Table in S1 File. Both authors independently assessed the risk of bias using Cochrane Risk of Bias Assessment Tool. Grading of Recommendations Assessment, Development and Evaluation (GRADE) tool was used to assess the uncertainty of evidence. Disagreements were discussed until consensus was reached.

### Data analysis

Key data needed for the review and meta-analysis was clearly identified in the source documents. Hence, authors of primary studies were not contacted. Quantitative data extracted from the source documents was the number of subjects in the intervention and control arms of the trial, and the number of SARS-CoV-2 infections in the two arms. Summary estimates were extracted from the source documents by both authors working independently. There was 100% agreement between the two authors regarding the number of infections in the vaccine and placebo groups. There were a few minor discrepancies between the two authors on the number of subjects in the vaccine and placebo groups in the trials. These were resolved by mutual discussion and with reference to the source documents. Janssen [14] reported separate results for eight trials, AstraZeneca [10, 11] reported separate results for four trials, and Novavax [16, 36] and Sinovac [37] reported results for two trials each. The multiple trials reported for a vaccine candidate do not include any overlapping subjects or observations across the trials. Hence, they are treated as separate data points in the meta-analysis [38]. Data points were not combined for the analysis. Duplicate data was not encountered in the source documents. Numbers associated with any sub-groups or sub-populations within a trial were not extracted or analysed. The numbers extracted were those reported for the overall trial. No trials whose efficacy results were available were excluded from the analysis.

The primary outcome and the measure of effect analysed was the risk ratio associated with each trial. The risk ratio was computed by the statistical software (STATA16.1) based on the number of infections and participants in the experimental and control arms of the trials. A random-effects model with restricted maximum likelihood estimator was employed in the meta-analysis. Heterogeneity between studies was assessed using the τ^2^ and *I*^*2*^ statistics [38]. The effect of pandemic prevalence on the variance in risk ratios across trials was assessed using the *R*^*2*^ statistic estimated by a meta-regression analysis.

Pandemic prevalence associated with a trial is estimated from the country-wise data on the SARS-CoV-2 positivity rates reported on Oxford University’s ‘Our World in Data’ portal [25]. The portal reports daily country-wise data for all countries, where available, on the number of people who tested positive for the SARS-CoV-2 infection in the country’s national SARS-CoV-2 testing program [25]. Daily SARS-CoV-2 positivity proportions corresponding to the duration of each trial were extracted from the portal. The prevalence of the SARS-CoV-2 pandemic associated with each trial was computed as the average of the daily positivity proportions prevailing over the duration of the trial (Table 2). Actual start dates for each trial were taken from the trials’ registration data on *ClinicalTrials*.*gov* and corroborated with the information reported in the peer reviewed documents, company reports and company press releases.

**Table 2:** Prevalence of the SARS-CoV-2 pandemic associated with each trial.

Vaccine candidate	Country of trial	Trial dates	Average daily SARS-CoV-2 prevalence rate (%)	Rank of pandemic prevalence^b^
AstraZeneca AZD1222	Brazil ^c^	April—Nov (2020)	63.1	16
AstraZeneca ^a^ AZD1222	US, Chile and Peru	Aug (2020)—March (2021)	7.88	7
AstraZeneca AZD1222 (LD/SD)	UK	April—Nov (2020)	4.8	4
AstraZeneca AZD1222 (SD/SD)	UK	April—Nov (2020)	4.8	4
Bharat Biotech, Covaxin	India	Nov (2020)—Feb (2021)	2.65	1
Gamaleya rAd26/rAd5	Russia	Sept—Nov (2020)	2.9	2
Janssen JNJ-78436735	Argentina	Sep (2020)—Jan (2021)	37.98	14
Janssen JNJ-78436735	Brazil ^c^	Sep (2020)—Jan (2021)	63.1	16
Janssen JNJ-78436735	Chile	Sep (2020)—Jan (2021)	14.84	11
Janssen JNJ-78436735	Columbia	Sep (2020)—Jan (2021)	22.1	13
Janssen JNJ-78436735	Mexico	Sep (2020)—Jan (2021)	41.53	15
Janssen JNJ-78436735	Peru	Sep (2020)—Jan (2021)	10.47	9
Janssen JNJ-78436735	South Africa	Sep (2020)—Jan (2021)	14.74	10
Janssen JNJ-78436735	United States	Sep (2020)—Jan (2021)	8.64	8
Moderna mRNA-1273	United States	July—Nov (2020)	6.31	5
Novavax NVX-CoV2373	South Africa	Nov (2020)—Jan (2021)	18.56	12
Novavax NVX-CoV2373	UK	Nov (2020)—Jan (2021)	7.3	6
Pfizer/BioNTech BNT162b2	United States	July—Nov (2020)	6.31	5
Sinovac CoronaVac	Brazil ^c^	July—Dec (2020)	63.1	16
Sinovac CoronaVac	Turkey	July—Dec (2020)	4.08	3

^a^ Average daily SARS-CoV-2 positivity rates in the US only was considered for this trial. The break-up of subjects across the three countries is not available, however, the number of centers running the trials in Chile and Peru was much smaller as compared to the number of centers in the US.

^b^ The lowest rank corresponds to the lowest ‘Average daily SARS-CoV-2 positivity rate’. Ties were awarded the same rank.

^c^ Daily raw SARS-CoV-2 positive rate data was not available for Brazil [25]. The World Health Organization’s website, https://worldhealthorg.shinyapps.io/covid/ mentions that in Week 37 of the pandemic (6^th^-12^th^ Sep 2020), the positivity rate in Brazil was 63.1%. This is substantially higher than the next highest rate (41.53 for Mexico, Rank 15). That enables us to assign the highest rank (16) to Brazil, even in the absence of specific data on Brazil’s positivity rate. This is consistent with all the other information on WHO’s website which shows that the pandemic in Brazil is far worse than that in any other country (https://worldhealthorg.shinyapps.io/covid/). It can also be inferred from Janssen [14] (Fig 10, p.55) that Brazil had the highest sustained rate of COVID-19 infections in the placebo group as compared to Argentina, Colombia, USA and South Africa over the period of the trial for which efficacy data reported, indicating that the prevalence of the pandemic in Brazil was higher than all the other countries.

The SARS-CoV-2 prevalence data across countries are not directly comparable due to the different protocols and practices across the different national SARS-CoV-2 testing programs [25]. Some of the key differences across countries include different testing regimes, different rates of testing, the different standards of testing, and different reporting practices across countries [25]. To address that limitation, we transformed the raw SARS-CoV-2 prevalence into ranks for use as a predictor in the meta-regression. While the raw SARS-CoV-2 positivity proportions may include a large error component, the ranks are likely to carry a much smaller error component.

## Results

**Fig 2:**
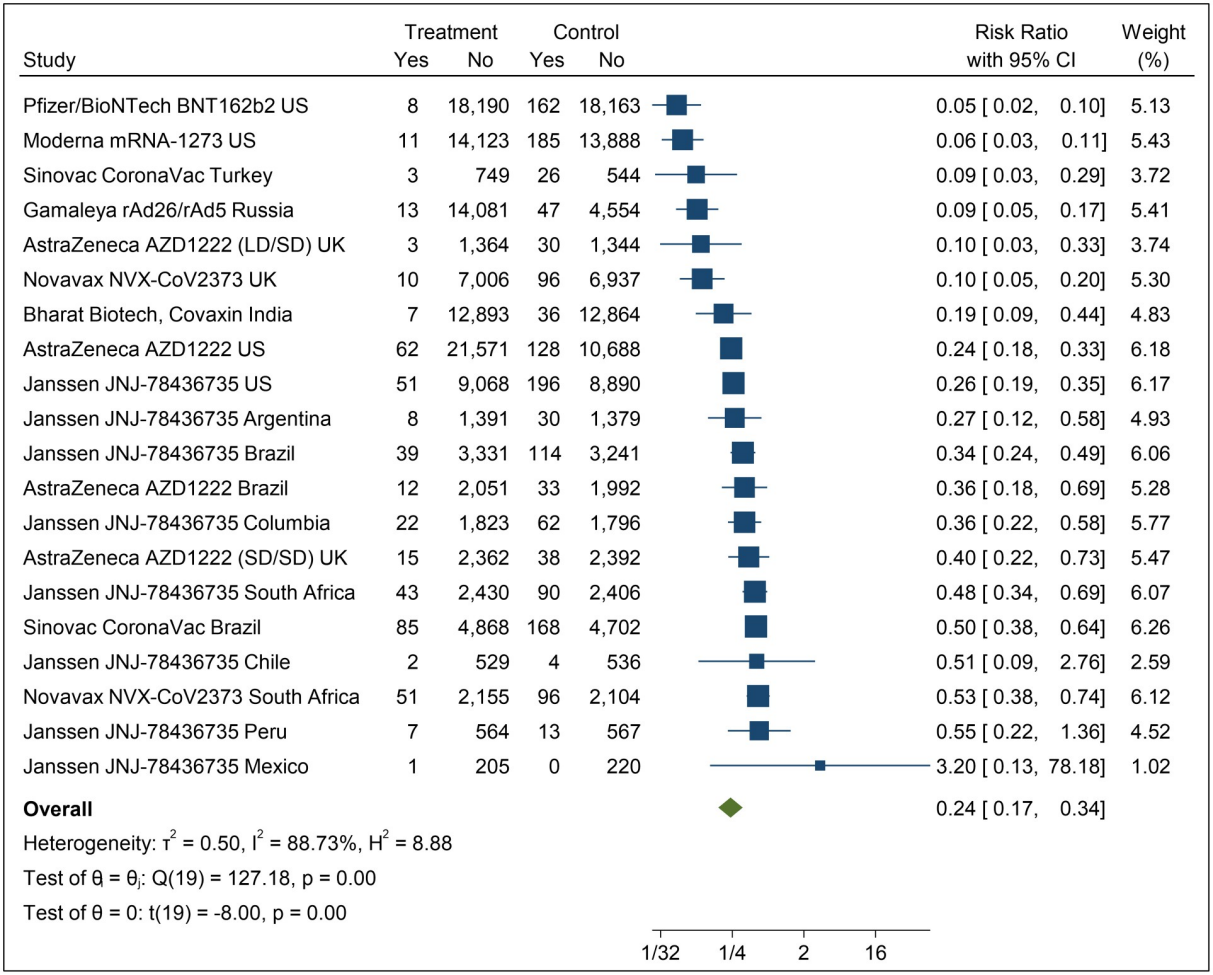
Forest plot.

**Fig 3:**
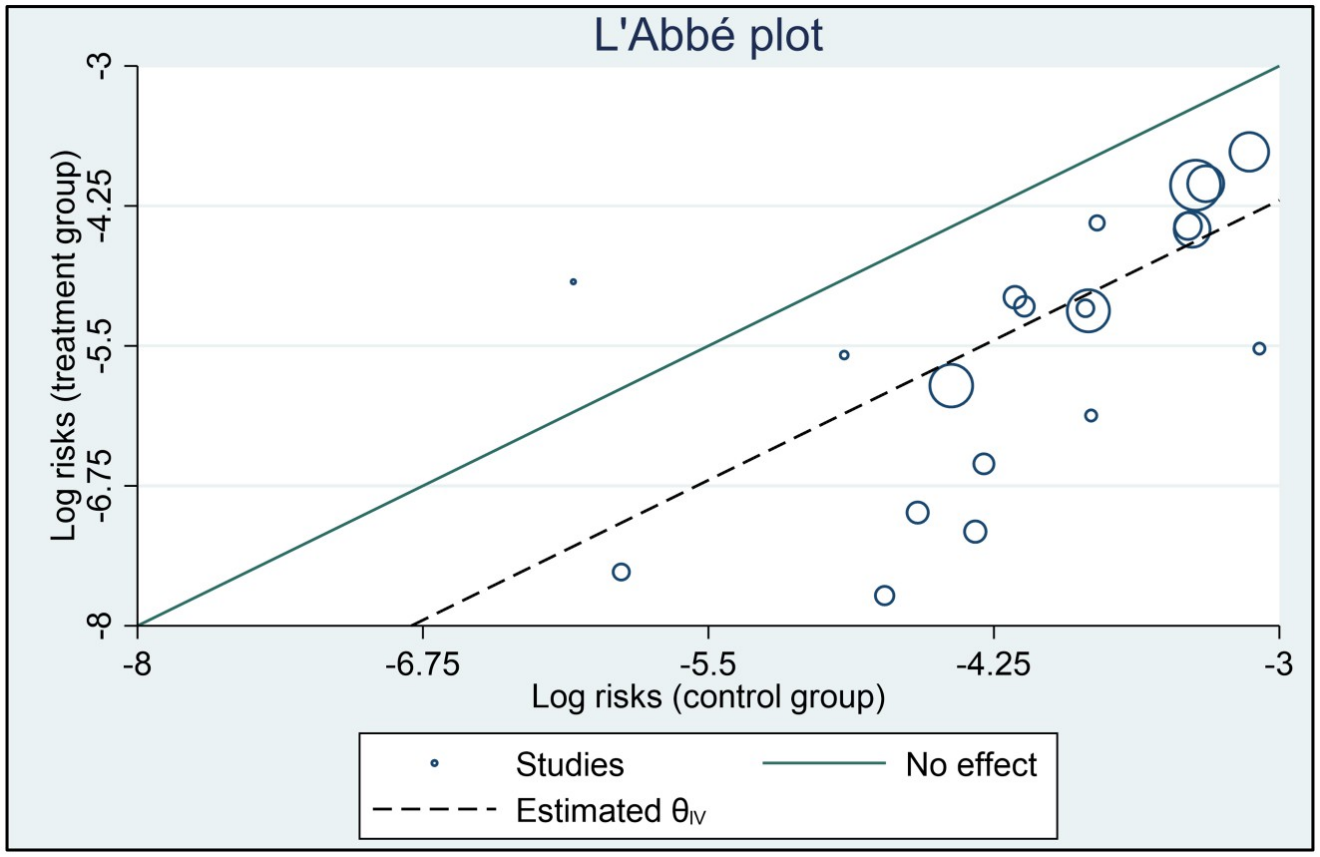
L’Abbe plot to explore heterogeneity.

The overall estimate of the risk-ratio (Forest plot reported in Fig 2) is 0.24 (p<0.01; 95% CI: 0.17, 0.34). The τ^2^ statistic is 0.50 and the I^2^ statistic is 88.73%, suggesting that the extent of heterogeneity is considerable, and that a high proportion of the total variance is due to between-study heterogeneity [39]. This justifies attempts to explain the heterogeneity using between-study differences which, in our case, is prevalence of the pandemic (L’Abbe plot to explore heterogeneity reported in Fig 3).

**Fig 4:**
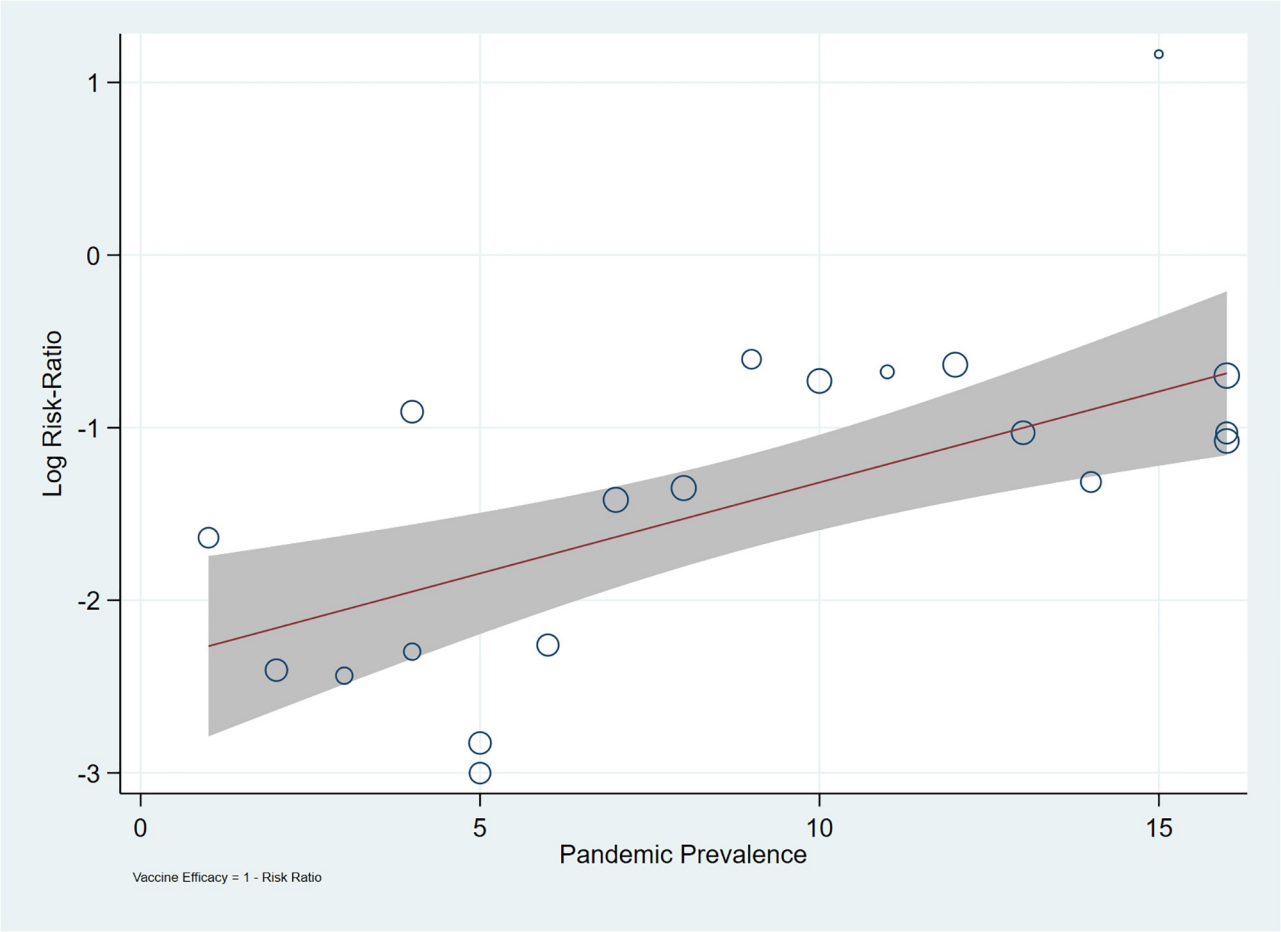
Association of pandemic prevalence on vaccine efficacy.

A meta-regression of the log risk ratio with pandemic prevalence as the moderator reports that almost half of the variance in log risk ratios across trials is explained by pandemic prevalence (R^2^ = 49.06%, p<0.01) (Table 3, Fig 4). The inverse relationship between pandemic prevalence and observed vaccine efficacy hypothesised in this study is supported.

**Table 3:** Results of meta-regression.

	Coefficient	Standard Error	t	P>t	[95%Conf. Interval]	
Pandemic prevalence	0.105	0.028	3.71	0.002	0.046	0.165
Constant	-2.371	0.291	-8.15	0.000	-2.982	-1.760

Note: Dependent Variable = Log Risk Ratio, Residual heterogeneity τ^2^ = 0.25, I^2^ = 79.3%, H^2^ = 4.83, R-squared = 49.06%, Test of residual homogeneity: Q_res = chi2(18) = 68.43, Prob>Q_res = 0.0000.

### Robustness checks

**Fig 5:**
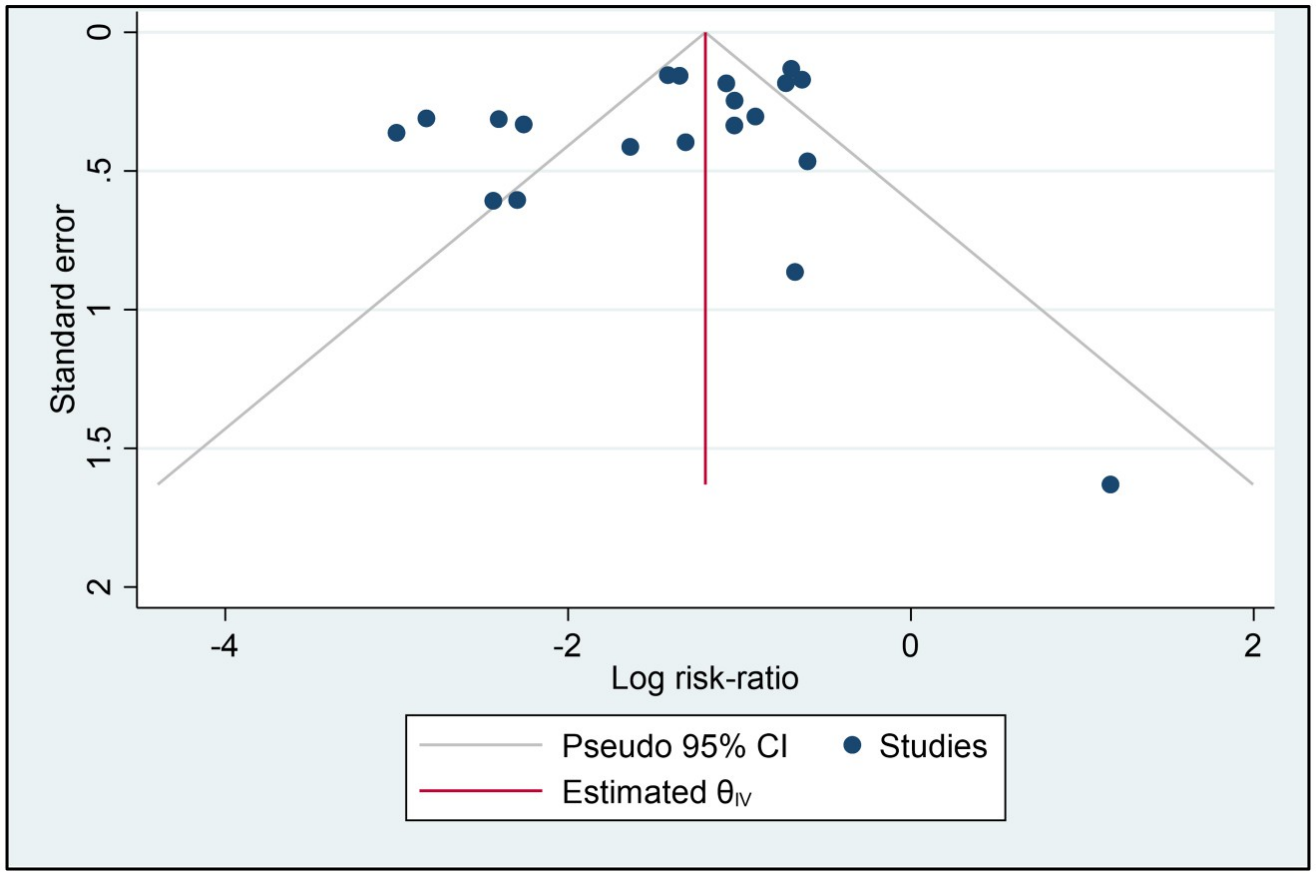
Funnel plot.

The funnel plot (See Fig 5) shows no visually obvious small-study effect on the findings. Results of the Egger test report no small-study effects on the findings (beta1 = 0.28, p = 0.73), supporting the visual interpretation (S4 Table in S1 File). Results of nonparametric trim-and-fill analysis of publication bias reports no evidence of publication bias due to missing studies; estimated number of imputed studies = 0 (S5 Table in S1 File).

Sensitivity analyses were conducted to analyse if the result of the meta-regression is sensitive to the inclusion of multiple trials for the Janssen vaccine (8 trials) or the AstraZeneca vaccine (4 trials). Two meta-regressions were run, one with an additional dummy variable for the Janssen vaccine, and the other with an additional dummy variable for the AstraZeneca vaccine. In both cases, the regression coefficient for pandemic prevalence was significant, while the coefficients for the dummy variable (Janssen/AstraZeneca) were non-significant (Tables 4 and 5). A separate meta-regression was also conducted including the 8 trials of the Janssen vaccine only; consistent with the results reported in Table 3, the results from this sub-sample also reported a significant effect of pandemic prevalence on vaccine efficacy.

**Table 4:** Results of meta-regression including JanssenJNJ as a dummy variable.

	Coefficient	Standard Error	t	P>t	[95%Conf. Interval]	
**Pandemic Prevalence**	0.091	0.032	2.85	0.011	0.024	0.159
**JannsenJNJ**	0.319	0.323	0.99	0.337	-0.362	1.10
**Constant**	-2.369	0.296	-7.98	0.000	-2.995	-1.742

Note: Dependent Variable = Log Risk Ratio, Residual heterogeneity τ^2^ = 0.27, I^2^ = 79.03%, H^2^ = 4.77, R-squared = 45.89%, Test of residual homogeneity: Q_res = chi2(17) = 67.71 Prob>Q_res = 0.0000.

**Table 5:** Results of meta-regression including AstraZeneca as a dummy variable.

	Coefficient	Standard Error	t	P>t	[95%Conf. Interval]	
**Pandemic Prevalence**	0.107	0.028	3.75	.002	0.047	0.168
**AstraZeneca**	0.267	0.345	0.78	0.449	-0.460	0.995
**Constant**	-2.447	0.308	-7.93	0.000	-3.098	-1.796

Note: Dependent Variable = Log Risk Ratio, Residual heterogeneity τ^2^ = 0.26, I^2^ = 78.81%, H^2^ = 4.72, R-squared = 48.14%, Test of residual homogeneity: Q_res = chi2(17) = 66.44 Prob>Q_res = 0.0000.

Another sensitivity analysis was run excluding the three trials conducted in Brazil. This was done as the daily SARS-CoV-2 positivity for Brazil was not available, consequently its rank of pandemic prevalence was estimated indirectly (see Table 2, Note c). Excluding those three trials does not change the result of the meta-regression; the effect of pandemic prevalence remains significant (R^2^ = 44.89%, p < 0.01) (Table 6).

**Table 6:** Results of meta-regression—Excluding three trials conducted in Brazil.

	Coefficient	Standard Error	t	P>t	[95%Conf. Interval]	
**Pandemic Prevalence**	0.137	0.041	3.29	0.005	0.048	0.227
**Constant**	-2.54	0.349	-7.27	0.000	-3.289	-1.798

Note: Dependent Variable = Log Risk Ratio, Residual heterogeneity τ^2^ = 0.30, I^2^ = 79.64%, H^2^ = 4.91, R-squared = 44.89%, Test of residual homogeneity: Q_res = chi2(15) = 55.2 Prob>Q_res = 0.0000.

Risk of bias was assessed as low using the Cochrane tool, while certainty of evidence was assessed as high using GRADE (S6 and S7 Tables in S1 File).

## Discussion

The meta-analysis (Forest plot, Fig 2) finds that the overall risk ratio across all 8 vaccines, reporting a total of 20 trials, is 0.24 (p<0.01; 95% CI: 0.17, 0.34), and includes a high degree of heterogeneity (τ^2^ = 0.50, I^2^ = 88.73%), attributable to between-study differences [8].

The results support our hypothesis that increasing pandemic prevalence is associated with lower efficacy of SARS-CoV-2 vaccine candidates (Table 3, R^2^ = 49.06%, p<0.05). Support for the hypothesis is robust, and not sensitive to potential sources of validity threats. The inclusion of multiple trials for the Janssen and AstraZeneca vaccine candidates does not affect the results of the meta-regression reported in Table 3. The finding reported in Table 3 is also robust against the inclusion of trials conducted in Brazil.

The key implication of this study is that a substantial proportion of the differences in efficacy observed across trials can be attributed to the prevalence of the SARS-CoV-2 pandemic across trial sites. This one source of heterogeneity explains almost 50% of the heterogeneity in efficacies observed across trials.

Table 3 reports that the residual heterogeneity (τ^2^) is 25% and the I^2^ statistic for the residual heterogeneity is 79.3%. This suggests that it is quite likely that the residual heterogeneity could be explained by additional between-trial differences. There are a number of other sources of heterogeneity that occur across Phase 3 trials of the SARS-CoV-2 vaccine candidates as well, including, but not limited to dosing protocols, interval between doses, and the definition of primary endpoints [22, 23, 29]. Future research could investigate the effect of other between-trial differences that could explain additional heterogeneity in observed efficacy.

The meta-regression analysis (Table 3) reports that vaccine efficacy falls as pandemic prevalence increases. A key implication of this finding is that the proper interpretation of efficacies reported by vaccine candidates needs to account for the effect of pandemic prevalence.

The finding that pandemic prevalence affects vaccine efficacy suggests three important implications for the conduct of Phase 3 trials of SARS-CoV-2 vaccine candidates. First, when efficacy results of Phase 3 trials are reported, they should be reported at the level of each trial site and include the level of pandemic prevalence associated with each trial site. Given the findings of this meta-analysis, a meaningful interpretation of observed efficacy in Phase 3 trials can only be done in conjunction with the associated data on pandemic prevalence across trial sites.

Second, it suggests that protocols for the design and conduct of Phase 3 trials need to take pandemic prevalence into account. The design of trials should include randomization across locations that are likely to vary in the prevalence of the pandemic. Further, for the duration of the Phase 3 trials, manufacturers should monitor pandemic prevalence at all locations where the trials are conducted. Results of Phase 3 trials should include an analysis of the effect of prevalence on vaccine efficacy.

Finally, the findings of this study suggests that Phase 3 trials should also analyse participant samples for the presence of mutant strains. Following prior research on the rotavirus and malaria vaccines, this study argues that the presence of mutant strains of the SARS-CoV-2 virus leads to both higher pandemic prevalence as well as a higher number of breakthrough infections. However, we are unable to directly test that mechanism as data on prevalent mutant strains at trial sites is not reported as part of the reporting protocols for Phase 3 trials. It is inevitable that there is a time lag between the sequencing of a mutant strain and assays to test for the presence of that strain. Hence, it is not always possible to report a mutant-specific analysis of the results of Phase 3 trials. However, the findings of this study suggest that this is an important relationship that has implications both for the development of vaccines as well as strategies for managing the pandemic. Accordingly, we suggest that protocols for Phase 3 trials should include the preservation of samples for the identification of mutant strains at various trial sites once the assays are available.

Overall, a key strength of this study is that it summarizes the entire current publicly available global evidence on the efficacy of SARS-CoV-2 vaccine candidates. It is also a strength of this study that it can explain almost half the variance in between-trial efficacies with one moderator variable only, pandemic prevalence. Another important strength of this meta-analysis is that data on pandemic prevalence is obtained from sources independent of the trials.

### Limitations

While this meta-analysis finds that, as hypothesized, higher pandemic prevalence is associated with lower vaccine efficacy, causal inferences need to be drawn with caution. As is the case with any meta-analysis, this is a post-hoc observational study. However, given the importance of the observed association, we suggest that future Phase 3 trials should employ protocols that allow this relationship to be tested via randomized controlled trials [9].

The overall sample is small, 20 trials across 8 vaccine candidates. Efficacy data on three other vaccine candidates that have completed Phase 3 trials and are currently approved for use are not publicly available and could not be included in this meta-analysis. All trials are multi-location, and a few are multi-country as well, e.g., Pfizer/BioNTech’s trial conducted in the US, Chile and Peru (see Table 2, Note a). In the absence of data on the exact locations of those trials and the number of subjects in the trial at each location, we have used country-level data to rank each trial on pandemic prevalence. However, since the trials were conducted in multiple locations across countries, using country-level data is justified. Nevertheless, this assumption does introduce an element of error in estimating pandemic prevalence across trial sites. To some extent, that effect is mitigated in our meta-regression by employing rank of pandemic prevalence as the moderator variable. Future Phase 3 trials could be required to report a location-level analysis to provide better insights into the efficacy results of Phase 3 trials.

## Conclusion

Pandemic prevalence has a significant negative effect on efficacies observed across Phase 3 trials of SARS-CoV-2 vaccine candidates; trials conducted in locations with low pandemic prevalence reported higher efficacies as compared to trials conducted in high pandemic prevalence locations.

## Supporting information

S1 File Supplement appendix with S1 to S7 Tables. (DOCX)

S2 File (DOCX)
