# Supplementary material for: The effect of pandemic prevalence on the reported efficacy of SARS-CoV-2 vaccines
Source: PLoS One. 2022 Apr 5;17(4):e0266271. doi: 10.1371/journal.pone.0266271 (PMC8982900; doi:10.1371/journal.pone.0266271)
Supplement: S1 File — (DOCX) [file pone.0266271.s001.docx]

**Supporting Information**

### S1 Table. Complete list of 26 vaccine candidates located in Phase 3 trials identified for this study

| **S. No.** | **26 vaccine candidates in Phase 3 trials identified for this study^a^**  **All vaccine candidates are listed in WHO [1] tracker, McGill tracker [2], and the LSHTM tracker [3]** |
| --- | --- |
| 1 | AnGes + Takara Bio + Osaka University |
| 2 | Anhui Zhifei Longcom Biopharmaceutical + Institute of Microbiology, Chinese Academy of Sciences |
| 3 | AstraZeneca + University of Oxford^b^ |
| 4 | Bharat Biotech International Limited |
| 5 | CanSino Biological Inc./Beijing Institute of Biotechnology |
| 6 | Center for Genetic Engineering and Biotechnology (CIGB) |
| 7 | Clover Biopharmaceuticals Inc./GSK/Dynavax |
| 8 | COVAXX + United Biomedical Inc |
| 9 | CureVac AG |
| 10 | Federal Budgetary Research Institution State Research Center of Virology and Biotechnology "Vector" |
| 11 | Gamaleya Research Institute; Health Ministry of the Russian Federation |
| 12 | Inovio Pharmaceuticals + International Vaccine Institute + Advaccine (Suzhou) Biopharmaceutical Co., Ltd |
| 13 | Institute of Medical Biology + Chinese Academy of Medical Sciences |
| 14 | Instituto Finlay de Vacunas |
| 15 | Janssen Pharmaceutical |
| 16 | Medicago Inc. |
| 17 | Moderna + National Institute of Allergy and Infectious Diseases (NIAID) |
| 18 | Novavax |
| 19 | Pfizer/BioNTech + Fosun Pharma |
| 20 | ReiThera + Leukocare + Univercells |
| 21 | Research Institute for Biological Safety Problems, Rep of Kazakhstan |
| 22 | Sanofi Pasteur + GSK |
| 23 | Shifa Pharmed Industrial Co |
| 24 | Sinopharm + China National Biotec Group Co + Beijing Institute of Biological Products |
| 25 | Sinopharm + China National Biotec Group Co + Wuhan Institute of Biological Products |
| 26 | Sinovac Research and Development Co., Ltd |
| ^a^ Names of vaccine candidates are as listed on WHO’s website *[1]*. The WHO website lists CSL Ltd. + Seqirus + University of Queensland as a vaccine candidate in Phase 3 trials. However, that vaccine has been withdrawn from development. The WHO site also lists Zydus Cadila as a vaccine candidate currently in Phase 3 trials. However, the Zydus Cadila vaccine candidate is currently in Phase 2 trials only (<http://ctri.nic.in/Clinicaltrials/showallp.php?mid1=45306&EncHid=&userName=Zydus>).  ^b^ McGill’s tracker includes a separate entry for Covishield, which is the name for AstraZeneca’s AZD1222 vaccine candidate that is being manufactured in India by the Serum Institute of India, and is registered in India for a Phase 3 trial under the name Covishield.  Apart from the minor discrepancies reported in a, and b above, there was 100% correspondence between the three sources regarding the 26 vaccine candidates in Phase 3 trials listed in Table S2 above.  AA independently verified the list of 26 vaccine candidates from the three sources *[1-3]* consulted by RS. AA also verified the list against vaccine candidates mentioned in recent reviews in the academic literature. A search of Google Scholar using the terms SARS-CoV-2 vaccines Phase 3 candidates and SARS-CoV-2 vaccines meta-analysis located a number of articles. Abstracts of two review articles suggested that they include a list of vaccine candidates in Phase 3 trials, and were chosen to corroborate the list of 26 vaccine candidates generated by RS. Specifically, Kyriakidis et al. (2021) *[4]* and Rogliani et al. (2021) *[5]* were selected. Kyriakidis et al. lists a total of 13 vaccine candidates currently in Phase 3 trials, all of which were included in the list of 26 generated by RS. Similarly, Rogliani et al.’s meta-analysis search identified a total of 17 vaccine candidates in Phase 3 trials; of those, they were able to locate Phase 2 trial data for 11 vaccine candidates. All the vaccine candidates named in Rogliani et al.’s meta-analysis were present in the list of 26 generated by RS. AA then referred to Wikipedia’s page on COVID-19 vaccines *[6]* to corroborate the list of 26 vaccine candidates. No additional vaccine candidates were located. | |

### S2 Table. Search for results of Phase 3 trials for the 26 vaccine candidates

| **Vaccine Developer** | **Results available on NCT register** | **Results available on company website** | **Company website searched (Outcome of search)** | **Result on Google search** |
| --- | --- | --- | --- | --- |
| AstraZeneca + University of Oxford | Y | Y | “76% vaccine efficacy against symptomatic COVID-19” (https://www.astrazeneca.com/media-centre/press-releases/2021/azd1222-us-phase-iii-primary-analysis-confirms-safety-and-efficacy.html) | “76% vaccine efficacy against symptomatic COVID-19” (https://www.bloomberg.com/press-releases/2021-03-25/azd1222-us-phase-iii-primary-analysis-confirms-safety-and-efficacy) |
| Bharat Biotech International Limited | N | Y | “Bharat Biotech Announces Phase 3 Results of COVAXIN®: India's First COVID-19 Vaccine Demonstrates Interim Clinical Efficacy of 81%.” (https://bharatbiotech.com/press_releases.html) | “India's Covaxin, the homegrown government-backed vaccine, has an efficacy rate of 81%, preliminary data from its phase 3 trial shows.” (https://www.bbc.com/news/world-asia-india-55748124) |
| Gamaleya Research Institute; Health Ministry of the Russian Federation | Y | N | No Information (https://gamaleya.org/en/research/vaktsina-protiv-covid-19/) | “The team found that starting 21 days after the first dose of the vaccine, the vaccine efficacy is 91.6 %” (<https://www.chemistryviews.org/details/news/11287590/Preliminary_Phase_3_Trial_Results_of_the_Russian_COVID-19_Vaccine_Sputnik_V.html_> “The first interim data analysis of the Sputnik V vaccine against COVID-19 phase III clinical trials in the Russian Federation demonstrated 92% efficacy”  (https://www.sciencemag.org/sites/default/files/The%20first%20interim%20data%20analysis%20of%20the%20Sputnik%20V%20vaccine%20against%20COVID-19%20phase%20III%20clinical%20trials%20in%20the%20Russian%20Federation%20demonstrated%2092%25%20efficacy%20.pdf) |
| Janssen Pharmaceutical | Y | Y | Covid19 status webpage (https://www.janssen.com/covid19) (https://www.janssen.com/johnson-johnson-announces-single-shot-janssen-covid-19-vaccine-candidate-met-primary-endpoints) | “…the vaccine was found to be 72 percent effective at preventing what the company defined as moderate to severe Covid-19” (https://www.nbcnews.com/health/health-news/how-effective-johnson-johnson-vaccine-what-know-n1259652) |
| Moderna + National Institute of Allergy and Infectious Diseases (NIAID) | Y | Y | (…Phase 3 Trial of the Moderna COVID-19 Vaccine…) (https://investors.modernatx.com/news-releases/news-release-details/moderna-announces-publication-results-pivotal-phase-3-trial) | “95% efficacy for COVID vaccines doesn’t mean there’s a 5% infection risk” (https://www.sacbee.com/news/coronavirus/article250559589.html) |
| Novavax | Y | Y | “Novavax COVID-19 Vaccine Demonstrates 89.3% Efficacy…” (https://ir.novavax.com/news-releases/news-release-details/novavax-covid-19-vaccine-demonstrates-893-efficacy-uk-phase-3) | “Novavax's (NVAX) COVID-19 Vaccine Shows Final Efficacy of 96.4%” (https://finance.yahoo.com/news/novavaxs-nvax-covid-19-vaccine-130301820.html) |
| Pfizer/BioNTech + Fosun Pharma | Y | Y | “Pfizer and Biontech announce publication of results from landmark phase 3 trial…” (https://www.pfizer.com/news/press-release/press-release-detail/pfizer-and-biontech-announce-publication-results-landmark) | “Pfizer and BioNTech have reported that updated topline results from Phase III study of their Covid-19 vaccine, BNT162b2, demonstrated an efficacy of 91.3% against the disease…” (https://www.pharmaceutical-technology.com/news/pfizer-biontech-vaccine-efficacy/) |
| Sinovac Research and Development Co., Ltd | Y | Y | “Summary of Clinical Trail Data of Sinovac’s COVID-19 Vaccine” (http://www.sinovac.com/?optionid=754&auto_id=927) | “A coronavirus vaccine developed by China's Sinovac has been found to be 50.4% effective…” (https://www.bbc.com/news/world-latin-america-55642648) |
| Sinopharm + China National Biotec Group Co + Wuhan Institute of Biological Products | N | N | No information (http://www.sinopharm.com/1156.html) | "No detailed efficacy data of Sinopharm’s COVID-19 vaccine has been publicly released" (https://www.reuters.com/article/us-health-coronavirus-who-china-vaccines-idUSKBN2BN1K8) |
| CanSino Biological Inc./Beijing Institute of Biotechnology | N | N | No information (http://www.cansinotech.com/) | "Cansino has not published its phase III trial data in a peer-reviewed journal, medicine's gold standard of vetting. In fact, all four Chinese vaccines on the market have failed to take this step" (https://fortune.com/2021/03/15/covid-vaccine-cansino-china-data/) |
| Sinopharm + China National Biotec Group Co + Beijing Institute of Biological Products | N | N | No information (http://www.sinopharm.com/1156.html) | "No detailed efficacy data of Sinopharm’s COVID-19 vaccine has been publicly released" (April 1, 2021) (https://www.reuters.com/article/us-health-coronavirus-who-china-vaccines-idUSKBN2BN1K8) |
| Federal Budgetary Research Institution State Research Center of Virology and Biotechnology "Vector" | N | N | No information, (http://www.vector.nsc.ru/) | "So far, however, researchers have released no corroborating data to the public — not in patents filed for the vaccine or in any other sources. Additionally, their claims about EpiVacCorona’s efficacy contradict the findings of an independent laboratory that analyzed blood samples from clinical-trial volunteers and found no neutralizing antibodies." (<https://meduza.io/en/feature/2021/03/23/epivaccorona-s-race-to-the-finish-line>)"The published research concerns EpiVacCorona’s combined Phase I and II trials (involving 14 and 86 volunteers, respectively), meaning that the data reflects only the drug’s short-term safety and side effects, as well as its ability to provoke an immune response in patients. These experiments weren’t designed to test the vaccine’s effectiveness, which can’t be assessed until the end of Phase III, which involves 3,000 participants." (https://meduza.io/en/feature/2021/03/27/sputnNik-v-s-ugly-cousin) |
| Medicago Inc. | N | N | "pleased to announce the start of Phase 3 clinical testing" https://www.medicago.com/en/media-room/medicago-and-gsk-start-phase-3-trial-of-adjuvanted-covid-19-vaccine-candidate/) | No Phase 3 results located |
| AnGes + Takara Bio + Osaka University | N | N | "Completion of inoculation in Phase 2/3 trial" (https://www.anges.co.jp/ir/) | No Phase 3 results located |
| Inovio Pharmaceuticals + International Vaccine Institute + Advaccine (Suzhou) Biopharmaceutical Co., Ltd | N | N | "In first quarter 2021, INOVIO completed enrollment of 400 subjects in the Phase 2 segment" (http://ir.inovio.com/news-releases/news-releases-details/2021/INOVIO-Reports-Fourth-Quarter-2020-and-Year-End-Financial-Results/default.aspx) | "completed enrolment of 400 participants in its Phase II segment" (https://www.biospace.com/article/inovio-expects-late-stage-covid-19-vaccine-trial-data-by-second-quarter/) |
| Sanofi Pasteur + GSK | N | N | "“We are pleased to be starting this new Phase 2 study … We look forward to further progressing this vaccine candidate to Phase 3 in Q2 2021" (https://www.sanofi.com/en/media-room/press-releases/2021/2021-02-22-11-40-00#) | No Phase 3 results located |
| CureVac AG | N | N | "trial will start with an initial Phase 2b part, which is expected to seamlessly merge into a Phase 3 efficacy trial" (https://www.curevac.com/en/2020/12/14/curevac-commences-global-pivotal-phase-2b-3-trial-for-covid-19-vaccine-candidate-cvncov/) | "Data from both clinical trials is expected in the second quarter of 2021" (https://www.pmlive.com/pharma_news/curevac_expands_covid-19_vaccine_trial_protocol_to_include_variant_specification_1365731) |
| Anhui Zhifei Longcom Biopharmaceutical + Institute of Microbiology, Chinese Academy of Sciences | N | N | No website located for Anhui | "There is no publicly available information in peer-reviewed scientific journals about the clinical trial data showing efficacy or safety." (https://www.thehindu.com/news/international/china-approves-fourth-covid-19-vaccine-for-emergency-use/article34080651.ece) |
| Research Institute for Biological Safety Problems, Rep of Kazakhstan | N | N | No website located | No Phase 3 results located |
| Institute of Medical Biology + Chinese Academy of Medical Sciences | - | - | - | Not searched. Trial start date later that 31 December 2020. |
| COVAXX + United Biomedical Inc | - | - | - | Not searched. Trial start date later than 31 December 2020. |
| Clover Biopharmaceuticals Inc./GSK/Dynavax | - | - | - | Not searched. Trial start date later than 31 December 2020. |
| Instituto Finlay de Vacunas | - | - | - | Not searched. Trial start date later than 31 December 2020. |
| Shifa Pharmed Industrial Co | - | - | - | Not searched. Trial start date later than 31 December 2020. |
| ReiThera + Leukocare + Univercells | - | - | - | Not searched. Trial start date later than 31 December 2020. |
| Center for Genetic Engineering and Biotechnology (CIGB) | - | - | - | Not searched. Trial start date later than 31 December 2020. |

### S3 Table. Primary data from Phase 3 Trials of SARS-CoV-2 vaccine candidates reporting efficacies

| **Vaccine Candidate** | **Population** | **Study type** | **Trial dates** | **Intervention** | **Primary endpoint for efficacy computation** | **Source** |
| --- | --- | --- | --- | --- | --- | --- |
| AstraZeneca AZD1222, Brazil (SD/SD) | 18+ years | Randomized, blinded, controlled trials | April – Nov (2020) | ChAdOx1 nCoV-19: two dose | “The primary end point was prevention of Covid-19 illness with onset at least 14 days after the second injection in participants who had not previously been infected with SARS-CoV-2” (p.104) | Voysey et al(2021) [7] |
| AstraZeneca AZD1222- US, Chile and Peru | 18+ years | Randomized, double-blind, placebo-controlled | Aug (2020) – March (2021) | ChAdOx1 nCoV-19: two dose | “The primary endpoint, vaccine efficacy at preventing symptomatic COVID-19 was 76% (confidence interval (CI): 68% to 82%) occurring 15 days or more after receiving two doses given four weeks apart.” | AstraZeneca. AZD1222 US Phase III primary analysis confirms safety and efficacy (https://www.astrazeneca.com/media-centre/press-releases/2021/azd1222-us-phase-iii-primary-analysis-confirms-safety-and-efficacy.html). March 25, 2021. |
| AstraZeneca AZD1222, UK (LD/SD) | 18+ years | Randomized, blinded, controlled trials | April – Nov (2020) | ChAdOx1 nCoV-19: two dose | “The primary end point was prevention of Covid-19 illness with onset at least 14 days after the second injection in participants who had not previously been infected with SARS-CoV-2” (p.104) | Voysey et al (2021) [7] |
| AstraZeneca AZD1222, UK (SD/SD) | 18+ years | Randomized, blinded, controlled trials | April – Nov (2020) | ChAdOx1 nCoV-19: two dose | “The primary end point was prevention of Covid-19 illness with onset at least 14 days after the second injection in participants who had not previously been infected with SARS-CoV-2” (p.104) | Voysey et al (2021) [7] |
| Bharat Biotech, COVAXIN, India | 18-98 years | Randomized, double-blind, Placebo-controlled | Nov (2020) – Feb (2021) | BBV152: two dose | “The primary endpoint of Phase 3 clinical trial is based on the first occurrence of PCR-confirmed symptomatic (mild, moderate, or severe)COVID-19 with onset at least 14 days after the second study vaccination in serologically negative (to SARS-CoV-2) adult participants at baseline.” | <https://www.bharatbiotech.com/images/press/covaxin-phase3-efficacy-results.pdf> |
| Gamaleya rAd26/rAd5, Russia | 18+ years | Randomised, double-blind, placebo-controlled | Sept – Nov (2020) | rAd26/rAd5: two dose | “The primary outcome was the proportion of participants with PCR-confirmed COVID-19 from day 21 after receiving the first dose.” (p.671, 675) | Logunov et al (2021) [8] |
| Janssen JNJ-78436735, Argentina | 18+ yearsor older | Randomized, double-blind, placebo-controlled | Sep (2020) – Jan (2021) | Ad26.COV2.S: 1dose | “First occurrence of molecularly confirmed, moderate to severe/critical COVID-19, with onset at least 14 days post-vaccination (Day 15)” (p.49) | Janssen Biotech Inc. (Feb 26, 2021) [9] |
| Janssen JNJ-78436735, Brazil | 18+ yearsor older | Randomized, double-blind, placebo-controlled | Sep (2020) – Jan (2021) | Ad26.COV2.S: 1dose | “First occurrence of molecularly confirmed, moderate to severe/critical COVID-19, with onset at least 14 days post-vaccination (Day 15)” (p.49) | Janssen Biotech Inc. (Feb 26, 2021) [9] |
| Janssen JNJ-78436735, Chile | 18+ yearsor older | Randomized, double-blind, placebo-controlled | Sep (2020) – Jan (2021) | Ad26.COV2.S: 1dose | “First occurrence of molecularly confirmed, moderate to severe/critical COVID-19, with onset at least 14 days post-vaccination (Day 15)” (p.49) | Janssen Biotech Inc. (Feb 26, 2021) [9] |
| Janssen JNJ-78436735, Columbia | 18+ yearsor older | Randomized, double-blind, placebo-controlled | Sep (2020) – Jan (2021) | Ad26.COV2.S: 1dose | “First occurrence of molecularly confirmed, moderate to severe/critical COVID-19, with onset at least 14 days post-vaccination (Day 15)” (p.49) | Janssen Biotech Inc. (Feb 26, 2021) [9] |
| Janssen JNJ-78436735, Mexico | 18+ yearsor older | Randomized, double-blind, placebo-controlled | Sep (2020) – Jan (2021) | Ad26.COV2.S: 1dose | “First occurrence of molecularly confirmed, moderate to severe/critical COVID-19, with onset at least 14 days post-vaccination (Day 15)” (p.49) | Janssen Biotech Inc. (Feb 26, 2021) [9] |
| Janssen JNJ-78436735, Peru | 18+ yearsor older | Randomized, double-blind, placebo-controlled | Sep (2020) – Jan (2021) | Ad26.COV2.S: 1dose | “First occurrence of molecularly confirmed, moderate to severe/critical COVID-19, with onset at least 14 days post-vaccination (Day 15)” (p.49) | Janssen Biotech Inc. (Feb 26, 2021) [9] |
| Janssen JNJ-78436735, South Africa | 18+ yearsor older | Randomized, double-blind, placebo-controlled | Sep (2020) – Jan (2021) | Ad26.COV2.S: 1dose | “First occurrence of molecularly confirmed, moderate to severe/critical COVID-19, with onset at least 14 days post-vaccination (Day 15)” (p.49) | Janssen Biotech Inc. (Feb 26, 2021) [9] |
| Janssen JNJ-78436735, US | 18+ yearsor older | Randomized, double-blind, placebo-controlled | Sep (2020) – Jan (2021) | Ad26.COV2.S: 1dose | “First occurrence of molecularly confirmed, moderate to severe/critical COVID-19, with onset at least 14 days post-vaccination (Day 15)” (p.49) | Janssen Biotech Inc. (Feb 26, 2021) [9] |
| Moderna mRNA-1273, US | 18+ yearsor older | Randomized, stratified, observer- blinded,placebo-controlled trial | July - Nov (2020) | mRNA-1273: two dose | “The primary end point was prevention of Covid-19 illness with onset at least 14 days after the second injection in participants who had not previously been infected withSARS-CoV-2.” (p.403) | Baden et al. (2021) [10] |
| Novavax NVX-CoV2373, South Africa | 18+ yearsor older | Randomized, observer- blinded,placebo-controlled trial | Nov (2020) – Jan (2021) | NVX-CoV2373; two dose | “Primary endpoint: PCR-positive symptomatic mild, moderate or severe COVID-19 illness diagnosed > 7 days after second dose” (Slide 26 of 45)[11] | Novavax [11-13] |
| Novavax NVX-CoV2373, UK | 18+ yearsor older | Randomized, observer- blinded,placebo-controlled trial | Nov (2020) – Jan (2021) | NVX-CoV2373; two dose | “Primary endpoint: PCR-positive symptomatic mild, moderate or severe COVID-19 illness diagnosed > 7 days after second dose” (Slide 20 of 45)[11] | Novavax [11-13] |
| Pfizer/BioNTech BNT162b2, US | 16+ or older | Randomized, observer- blinded,placebo-controlled trial | July - Nov (2020) | BNT162b2 mRNA: two dose | “The time period for Covid-19 case accrual is from 7 days after the second dose to the end of the surveillance period.” (p.2612) | Polack et al. (2020) [14] |
| Sinovac CoronaVac, Brazil | 18+ years | Randomized, double-blind, and placebo-controlled | July – Dec (2020) | CoronaVac: two dose | “14 Days after 2 Doses of Vaccination in Phase III Clinical Trial” (Table 3, 4)[15] | Sinovac [15] |
| Sinovac CoronaVac, Turkey | 18 to 59 | Randomized, double-blind, and placebo-controlled | July – Dec (2020) | CoronaVac: two dose | “14 Days after 2 Doses of Vaccination in Phase III Clinical Trial” (Table 3, 4)[15] | Sinovac [15] |

| S4 Table. Results of Meta-regression –Egger Test | | | | | | |
| --- | --- | --- | --- | --- | --- | --- |
|  | **Coefficient** | **Standard Error** | **t** | **P>t** | **[95%Conf. Interval]** | |
| **Meta_se** | 0.278 | 0.786 | 0.35 | 0.728 | -1.380 | 1.936 |
| **Pandemic Prevalence** | 0.108 | 0.030 | 3.59 | 0.002 | 0.045 | 0.172 |
| **Constant** | -2.482 | 0.426 | -5.82 | 0.000 | -3.381 | -1.583 |
| Note: Dependent Variable = Log Risk Ratio, Residual heterogeneity I^2^ = 80.81%, H^2^ = 5.21, R^2^ = 44.75%, Test of residual homogeneity: Q_res = chi2(17) = 65.86 Prob>Q_res = 0.0000,H0: beta1 = 0; no small-study effectsbeta1 (no small study effect) = 0.28Standard Error of beta1 = 0.786t = 0.35Prob>\|t\| = 0.73 | | | | | | |

| S5 Table. Non-parametric trim and fill test | | |
| --- | --- | --- |
|  | **Log Risk-Ratio** | **[95%Conf. Interval]** |
| **Observed** | -1.423 | -1.776 -1.070 |
| **Observed + Imputed** | -1.423 | -1.776 -1.070 |
| Number of studies = 20, observed = 20, imputed = 0 | | |

| S6 Table. Risk of Bias Assessment |
| --- |
| **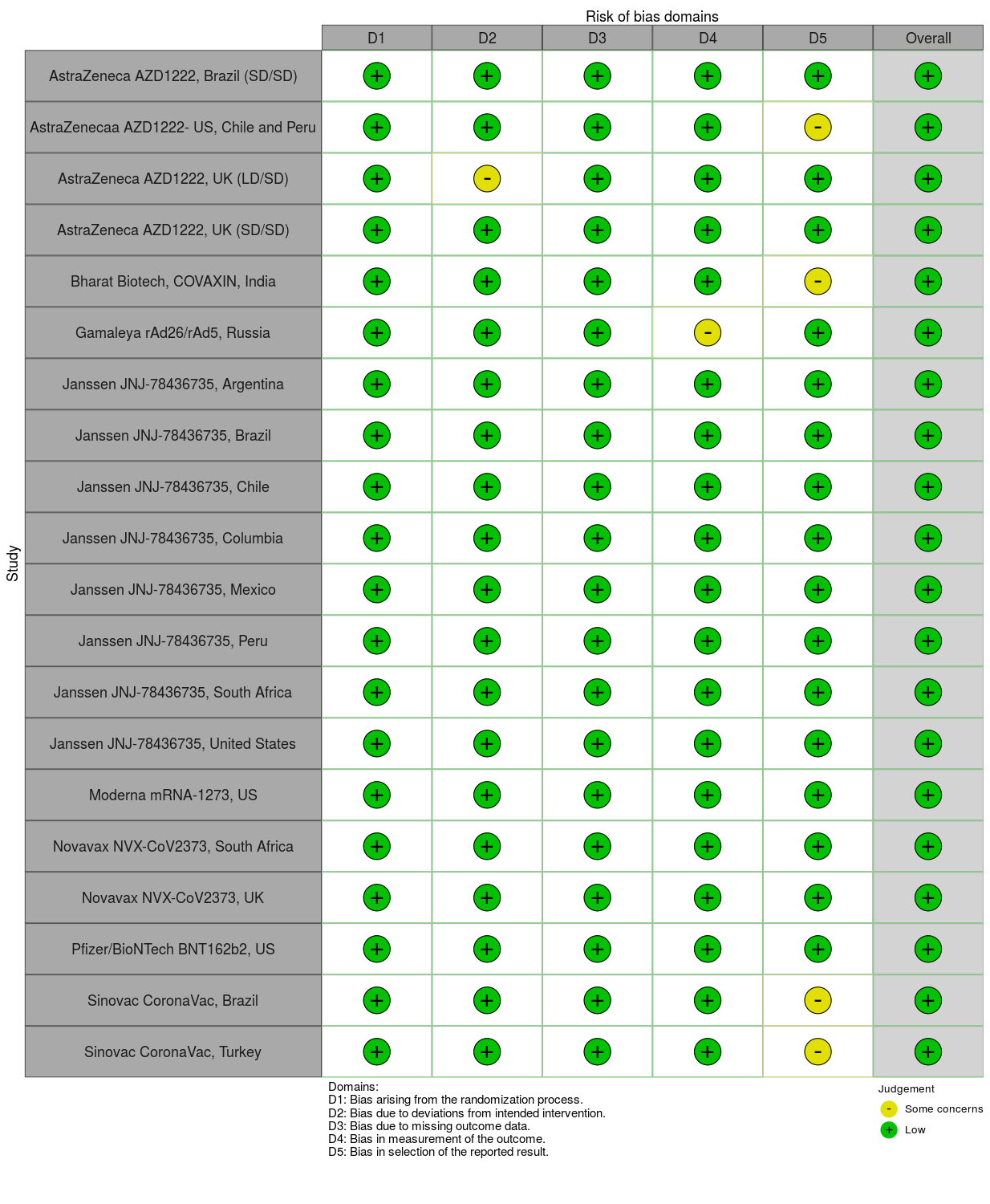** Notes:  1. The AstraZeneca (UK, LD/SD) trial deviated from the original protocol. The issues are addressed in Voysey et al (2021)[7] and have been peer reviewed. That deviation from protocol does not make a material difference to the overall risk of bias assessment as all other aspects of RCT protocol were adhered to.  2. Gamaleya deviated from the original protocol by changing the primary outcome of the trial midway through the trial. Specifically, the end point was taken as 21 days after the first dose, rather than 14 days after the second dose in the original protocol. The issue has been reported in Logunov et al (2021)[8] and has been peer reviewed. All other aspects of RCT were per protocol. The deviation from protocol does not make a material difference to the overall risk of bias assessment.  3. For AstraZeneca (US, Chile and Peru trial), Bharat Biotech and Sinovac, the data on primary outcomes are taken from the company press releases. Results from their Phase 3 trials have not been publicly reported or peer reviewed. However, both vaccines had their Phase 3 trial protocols approved by their respective regulatory authorities. Further, all three vaccines have been approved for use by various national authorities. The overall risk of bias is considered as low. |

| S7 Table. GRADE Assessment | | | | | | | | | | | | |
| --- | --- | --- | --- | --- | --- | --- | --- | --- | --- | --- | --- | --- |
| **Quality Assessment** | | | | | | | **No of Subjects** | | **Effect** | | **Quality** | **Importance** |
| No of Studies | Design | Risk of Bias | Inconsistency | Indirectness | Imprecision | Other Considerations | Treatment (Vaccine Group) | Control (Placebo Group) | Relative (95% CI) | Absolute Risk Difference |  |  |
| 4 (peer reviewed) | RCT | No serious risk of bias | No serious inconsistency | No serious indirectness | None | None | 62/52,233 (0.12%) | 495/42,828 (1.16%) | RR: 0.13 (0.05, 0.34) | -0.011 (-0.013, -0.008) | High | Critical |
| 8 (all sources, including not peer reviewed) | RCT | No serious risk of bias | No serious inconsistency | No serious indirectness | None | Sources not peer reviewed^a^ | 453/121,207 (0.37%) | 1554/100,761 (1.54%) | RR: 0.24 (0.17, 0.34) | -0.013 (-0.016, -0.009) | High | Critical |
|  |  |  |  |  |  |  |  |  |  |  |  |  |
| Note: ^a^Results of two Phase 3 trials reported by Sinovac, and one each by Bharat Biotech, and the AstraZeneca (US, Chile, Peru trial) were not peer reviewed. However, as per the GRADE assessment above (eTable 9), the overall risk of bias for the non-peer reviewed studies is assessed to be low. | | | | | | | | | | | | |

References

1. WHO. Draft landscape and tracker of COVID-19 candidate vaccines 2021 [Available from: <https://www.who.int/emergencies/diseases/novel-coronavirus-2019/covid-19-vaccines>.

2. McGill University COVID19 Vaccine Tracker Team. COVID19 Vaccine Tracker website 2021 [Available from: <https://covid19.trackvaccines.org/>.

3. Shrotri S, Kampmann P. An interactive website tracking COVID-19 vaccine development. The Lancet Global Health. 2021;9(5):E590-E2.

4. Kyriakidis NC, López-Cortés A, González EV, Grimaldos AB, Prado EO. SARS-CoV-2 vaccines strategies: a comprehensive review of phase 3 candidates. npj Vaccines. 2021;6(1):1-17.

5. Rogliani P, Chetta A, Cazzola M, Calzetta L. SARS-CoV-2 Neutralizing Antibodies: A Network Meta-Analysis across Vaccines. Vaccines. 2021;9(3):227.

6. Wikipedia(c). COVID-19 vaccine Last Update 28 March 2021, at 15:27 (UTC), [Available from: <https://en.wikipedia.org/wiki/COVID-19_vaccine>.

7. Voysey M, Clemens SAC, Madhi SA, Weckx LY, Folegatti PM, Aley PK, et al. Safety and efficacy of the ChAdOx1 nCoV-19 vaccine (AZD1222) against SARS-CoV-2: an interim analysis of four randomised controlled trials in Brazil, South Africa, and the UK. The Lancet. 2021;397(10269):99-111.

8. Logunov DY, Dolzhikova IV, Shcheblyakov DV, Tukhvatulin AI, Zubkova OV, Dzharullaeva AS, et al. Safety and efficacy of an rAd26 and rAd5 vector-based heterologous prime-boost COVID-19 vaccine: an interim analysis of a randomised controlled phase 3 trial in Russia. The Lancet. 2021;397(10275):671-81.

9. Sponsor briefing document - vaccines and related biological products advisory committee meeting date: 26 February 2021 [press release]. March 26, 2021 2021.

10. Baden LR, El Sahly HM, Essink B, Kotloff K, Frey S, Novak R, et al. Efficacy and Safety of the mRNA-1273 SARS-CoV-2 Vaccine. New England Journal of Medicine. 2020;384(5):403-16.

11. Glenn G. Efficacy Data Updates from Novavax’ Protein-based Vaccine Candidate. Presentation to the New York Academy of Sciences symposium, “The Quest for a COVID-19 Vaccine”, ; Feb 2-3, 2021 February 2, 2021.

12. Novavax COVID-19 Vaccine Demonstrates 89.3% Efficacy in UK Phase 3 Trial (<https://ir.novavax.com/news-releases/news-release-details/novavax-covid-19-vaccine-demonstrates-893-efficacy-uk-phase-3>) [press release]. Jan 28, 2021.

13. Novavax. Novavax Confirms High Levels of Efficacy Against Original and Variant COVID-19 Strains in United Kingdom and South Africa Trials 2021 [Available from: <https://ir.novavax.com/news-releases/news-release-details/novavax-confirms-high-levels-efficacy-against-original-and-0>.

14. Polack FP, Thomas SJ, Kitchin N, Absalon J, Gurtman A, Lockhart S, et al. Safety and efficacy of the BNT162b2 mRNA Covid-19 vaccine. New England Journal of Medicine. 2020;383(27):2603-15.

15. Summary of Clinical Trail Data of Sinovac’s COVID-19 Vaccine (CoronaVac®) (<http://www.sinovac.com/?optionid=754&auto_id=927>) [press release]. April 3, 2021.
