# Supplementary material for: The effect of pandemic prevalence on the reported efficacy of SARS-CoV-2 vaccines
Source: PLoS One. 2022 Apr 5;17(4):e0266271. doi: 10.1371/journal.pone.0266271 (PMC8982900; doi:10.1371/journal.pone.0266271)
Supplement: S2 File — (DOCX) [file pone.0266271.s002.docx]

| Vaccine candidate | Number of subjects in vaccine group | Number of infections in vaccine group | Number of subjects in placebo group | Number of infections in placebo group | Reported efficacy (%) | Trial dates | Average daily SARS-CoV-2 prevalence rate (%) | Rank of pandemic prevalence |
| --- | --- | --- | --- | --- | --- | --- | --- | --- |
| AstraZeneca AZD1222, Brazil (SD/SD) | 2063 | 12 | 2025 | 33 | 64.2 | April (2020) – Nov (2020) | 63.1 | 16 |
| AstraZeneca AZD1222- US, Chile and Peru | 21633 | 62 | 10816 | 128 | 76 | Aug (2020) – March (2021) | 7.88 | 7 |
| AstraZeneca AZD1222, UK (LD/SD) | 1367 | 3 | 1374 | 30 | 90 | April (2020) – Nov (2020) | 4.8 | 4 |
| AstraZeneca AZD1222, UK (SD/SD) | 2377 | 15 | 2430 | 38 | 60.3 | April (2020) – Nov (2020) | 4.8 | 4 |
| Bharat Biotech, COVAXIN, India | 12900 | 7 | 12900 | 36 | 80.6 | Nov (2020) – Feb (2021) | 2.65 | 1 |
| Gamaleya rAd26/rAd5, Russia | 14094 | 13 | 4601 | 47 | 91.1 | Sept 2020) – Nov (2020) | 2.9 | 2 |
| Janssen JNJ-78436735, Argentina | 1399 | 8 | 1409 | 30 | 73.3 | Sep (2020) – Jan (2021) | 37.98 | 14 |
| Janssen JNJ-78436735, Brazil | 3370 | 39 | 3355 | 114 | 66.2 | Sep (2020) – Jan (2021) | 63.1 | 16 |
| Janssen JNJ-78436735, Chile | 531 | 2 | 540 | 4 | 49.6 | Sep (2020) – Jan (2021) | 14.84 | 11 |
| Janssen JNJ-78436735, Columbia | 1845 | 22 | 1858 | 62 | 64.7 | Sep (2020) – Jan (2021) | 22.1 | 13 |
| Janssen JNJ-78436735, Mexico | 206 | 1 | 220 | 0 | - | Sep (2020) – Jan (2021) | 41.53 | 15 |
| Janssen JNJ-78436735, Peru | 571 | 7 | 580 | 13 | 45.3 | Sep (2020) – Jan (2021) | 10.47 | 9 |
| Janssen JNJ-78436735, South Africa | 2473 | 43 | 2496 | 90 | 52 | Sep (2020) – Jan (2021) | 14.74 | 10 |
| Janssen JNJ-78436735, United States | 9119 | 51 | 9086 | 196 | 74.4 | Sep (2020) – Jan (2021) | 8.64 | 8 |
| Moderna mRNA-1273, US | 14134 | 11 | 14073 | 185 | 94.1 | July (2020) - Nov (2020) | 6.31 | 5 |
| Novavax NVX-CoV2373, South Africa | 2206 | 51 | 2200 | 96 | 48.6 | Nov (2020) – Jan (2021) | 18.56 | 12 |
| Novavax NVX-CoV2373, UK | 7016 | 10 | 7033 | 96 | 96.4 | Nov (2020) – Jan (2021) | 7.3 | 6 |
| Pfizer/BioNTech BNT162b2, US | 18198 | 8 | 18325 | 162 | 95 | July (2020) - Nov (2020) | 6.31 | 5 |
| Sinovac CoronaVac, Brazil | 4953 | 85 | 4870 | 168 | 50.65 | July (2020) – Dec (2020) | 63.1 | 16 |
| Sinovac CoronaVac, Turkey | 752 | 3 | 570 | 26 | 91.25 | July (2020) – Dec (2020) | 4.08 | 3 |
